# Comparing the outcomes of termination of second trimester pregnancy with a live fetus using intravaginal misoprostol between women with and without previous cesarean section

**DOI:** 10.1186/s12884-024-06442-x

**Published:** 2024-04-12

**Authors:** Saipin Pongsatha, Nuchanart Suntornlimsiri, Theera Tongsong

**Affiliations:** Department of Obstetrics and Gynecology, Faculty of Medicine, Chiang Mai University, Chiang Mai, 50200 Thailand

**Keywords:** Cesarean section, Live fetus, Misoprostol, Second trimester, Termination of pregnancy

## Abstract

**Objective:**

To compare the outcomes of termination of pregnancy with live fetuses in the second trimester (14–28 weeks), using misoprostol 400 mcg intravaginal every 6 h, between women with previous cesarean section (PCS) and no previous cesarean section (no PCS).

**Methods:**

A comparative study was conducted on a prospective database of pregnancy termination in the second trimester, Chiang Mai university hospital. Inclusion criteria included: (1) singleton pregnancy; (2) gestational age between 14 and 28 weeks; and (3) pregnancy with a live fetus and medically indicated for termination. The participants were categorized into two groups; PCS and no PCS group. All were terminated using misoprostol 400 mcg intravaginal every 6 h. The main outcomes were induction to fetal delivery interval and success rate, defined as fetal delivery within 48 h.

**Results:**

A total of 238 women, including 80 PCS and 158 no PCS, were recruited. The success rate of fetal delivery within 48 h between both groups was not significantly different (91.3% vs. 93.0%; p-value 0.622). The induction to fetal delivery interval were not significantly different (1531 vs. 1279 min; p-value > 0.05). Gestational age was an independent factor for the success rate and required dosage of misoprostol. The rates of most adverse effects of misoprostol were similar. One case (1.3%) in the PCS group developed uterine rupture during termination, ending up with safe and successful surgical removal and uterine repair.

**Conclusion:**

Intravaginal misoprostol is highly effective for second trimester termination of pregnancy with PCS and those with no PCS, with similar success rate and induction to fetal delivery interval. Gestational age was an independent factor for the success rate and required dosage of misoprostol. Uterine rupture could occur in 1.3% of PCS, implying that high precaution must be taken for early detection and proper management.

**Synopsis:**

Intravaginal misoprostol is highly effective for termination of second trimester pregnancy with a live fetus, with a comparable success rate between women with and without previous cesarean section, with a 1.3% risk of uterine rupture among women with previous cesarean section.

## Introduction

Currently, the cesarean section (CS) rate tends to increase worldwide. However, there is very high and significant heterogeneity among countries, which follow independent pattern [1]. The CS rate is greatest in Latin America (52%) [2], followed by USA (32.1%) [3]. The CS rate is higher in urban areas than that in rural areas [1], and much higher in private hospitals [4], possibly as high as 75% in some hospitals [5]. In Thailand, the overall CS rate has been increasing from 15.2% in 1990 to 32.5 in 2017 [6]. As a consequence, the number of cases with termination of pregnancy (TOP) with previous cesarean section (PCS) in the second trimester has been increasing, leading to more challenging in management since PCS is well recognized as one of the most dangerous risk of uterine rupture during the process of TOP.

TOP by misoprostol is one of the most popular methods because of high effectiveness, low cost, simplicity for use with various routes of drug administration, and minimal adverse effects. The adverse effect of most concern is uterine rupture, especially in cases with PCS, which has been markedly increasing as mentioned above. The studies on misoprostol as a single medication for TOP in this group of women in the second trimester are very limited. Moreover, such studies were heterogeneous in terms of dosage, route and interval of misoprostol regimens and fetal live status [7–10]. Based on the limited data, outcomes of TOP with misoprostol were similar between the women with PCS and with no PCS [8, 10]. Of note, in the studies of TOP with death fetus, the termination outcomes were more favorable in the pregnancies with no PCS [9]. Uterine rupture associated with misoprostol use was reported to be less than 1% (0.78%) in women with one PCS [10], but up to 11.5% in the cases of at least two PCSs [11]. Additionally, some recent studies prefer using mifepristone and then followed by intravaginal misoprostol because of a higher success rate and shorter fetal delivery interval, when compared to misoprostol alone [12]. However, because mifepristone is of higher cost, and not worldwide available, TOP in the second trimester with misoprostol alone is much more commonly used in clinical practice.

According to WHO recommendations for medical management of abortion [13], for those with uterine scars, the safety and efficacy of medical abortion regimens is an area requiring more research, especially the misoprostol dosage. Because of limited and heterogeneous data concerning misoprostol used in TOP with PCS in the second trimester, in terms of various misoprostol regimens and fetal life status, we conducted this study, primarily aimed to compare the effectiveness and adverse effect of intravaginal misoprostol 400 mcg every 6 h for TOP with live fetuses in the second trimester between pregnancies with PCS and those with no PCS.

## Patients and methods

The comparative study was conducted, based on the prospective database of second trimester termination of pregnancy (TOP) of Obstetrics and Gynecology Department (Maharaj Nakorn Chiang Mai Hospital), Chiang Mai University, Thailand. The database of TOP was developed in 1997 and since then has prospectively collected data about misoprostol use for TOP in the second trimester. The present study was ethically approved by the Institutional Review Boards, Faculty of Medicine, Chiang Mai University (Research Study ID 08184). The inclusion criteria for the study group (pregnancy with previous cesarean; PCS group) are as follows: (1) singleton pregnancy; (2) gestational age between 14 and 28 weeks; (3) live fetus with an indication for therapeutic termination such as major fetal anomaly or fetal severe fetal thalassemia etc.; 3) history of at least one previous CS; (4) unfavorable cervix, defined as Bishop score of four or less; and (5) having no labor pain prior to induction. The inclusion criteria for the control group (pregnancy with no previous cesarean; no PCS group) are the same, except no history of PCS). The exclusion criteria are as follows: (1) contraindication of vaginal delivery such as placenta previa totalis or placenta accreta spectrum disorder, etc.; (2) Membranes rupture prior to misoprostol administration; (3) hypersensitivity to misoprostol. After counseling, all participants received the same regimen of misoprostol as a single medication (400 mcg intravaginal every 6 h). Note that, typically the regimen for termination of a live fetus in the second trimester is 400 mcg in transvaginal every 3 h, but because of theoretically higher risk of uterine rupture secondary to previous uterine scar, in this study we used 6-hour interval of misoprostol administration. To diminish the variation of misoprostol regimen in the present study, therefore we used the same regimen (400 mcg intravaginal every 6 h) in both groups.

Baseline maternal demographic and clinical characteristics and TOP outcomes were prospectively recorded, including maternal age, parity, gestational age, the number of previous cesarean sections, indication for TOP, Bishop score just prior to misoprostol administration, induction to fetal and placental delivery interval, the rate of complete fetal delivery within 12, 24, 36 and 48 h after the initiation of misoprostol, failure to fetal delivery within 48 h after the initiation of misoprostol, oxytocin and intravenous analgesia requirement, estimated blood loss postpartum hemorrhage, total number and total dose of misoprostol used, the number of cases requiring oxytocin use. Misoprostol adverse effects such as fever, chill, diarrhea, uterine laceration or rupture were also prospectively recorded. The successful termination was defined as fetal delivery within 48 h. The primary outcomes were the success rate of termination and induction to fetal / placental delivery time interval.

In TOP process, after administration of the first dose (400 mcg), the same dose of intravaginal misoprostol was repeated every 6 h if the Bishop score was still of four or less and uterine contractions could not regularly be achieved. Nevertheless, in cases the cervix was unfavorable but adequate uterine contraction was present, the next scheduled dose of misoprostol was skipped and reassessed again at the following scheduled time interval. If adequate uterine contraction was not achieved and the cervix was still unfavorable, then intravaginal misoprostol was repeated again. If the cervix was progressive to be favorable (Bishop score of five or more) but adequate uterine contractions were not achieved, misoprostol at the scheduled time interval was discontinued and intravenous oxytocin was instead given by automatic infusion pump, starting with 2 milliunits per min and increased as necessary, every 15 min to 4, 8, 12, 16, 20, 25, and maximum of 30 milliunits per min. Pethidine 50 mg was given intravenously for painful uterine contraction, as requested by the participants. All were taken a standard of care as a high risk patient with close surveillance of maternal vital signs as well as signs of uterine rupture and availability of an operation room, obstetricians and anesthetists in case of emergency.

### Statistical analysis

Statistical procedures were performed using the statistical package for the social sciences (SPSS) software version 26.0 (IBM Corp. Released 2019. IBM SPCSS Statistics for Windows, Armonk, NY: IBM Corp). The baseline data were presented as mean ± SD or median (IQR) for continuous data as suitable, and as percentage for categorical data. In the comparisons, Chi-square test was performed for the categorical data, whereas Student’s test or Mann-Whitney-U was conducted for the continuous data, as appropriate. A *p*-value of less than 0.05 was defined as statistical significance.

## Results

During the study period, a total of 238 pregnant women met the inclusion criteria and available for analysis, consisting of 80 in PCS group and 158 in No PCS group. Most maternal baseline characteristics were comparable between both groups. Nevertheless, women in PCS group had significantly higher mean maternal age and higher parity, as presented in Table 1. The most common indications for TOP were fetal severe thalassemia, chromosomal abnormalities and serious structural anomalies.

**Table 1:** Baseline maternal characteristics and peripartum variables

	Previous cesarean Sect. (80)	No previous cesarean Sect. (158)	P value
Age (yr)	34.49 ± 5.42	30.59 ± 7.01	< 0.001
Pre-pregnancy BMI	23.58 ± 9.14	23.24 ± 8.71	0.890
Gestational age (wk)	19.51 ± 2.89	20.22 ± 3.05	0.086
Parity			< 0.001
Nulliparous women: n (%)	0 (0.0%)	100 (63.3%)	
Parous women: n (%)	80 (100.0%)	58 (36.7%)	
Bishop score	1.14 ± 0.99	1.36 ± 0.97	0.096
Indication for termination			0.407
Severe thalassemia: n (%)	23 (28.8%)	64 (40.5%)	
Chromosome abnormality: n (%)	25 (31.3%)	35 (22.2%)	
Severe anomaly: n (%)	27 (33.8%)	48 (30.4%)	
Maternal complication: n (%)	1 (1.3%)	2 (1.3%)	
Others: n (%)	4 (5.0%)	9 (5.7%)	

**Fig. 1:**
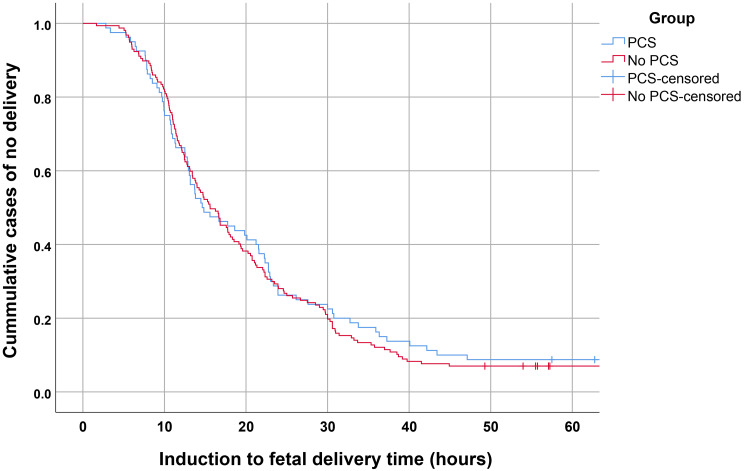
Kaplan-Meier curves for the rates of no delivery of the groups of PCS and No PCS as a function of induction to fetal delivery time (Log rank test; p-value 0.664)

In comparisons of termination of pregnancy outcomes, the success rate of fetal delivery within 48 h between both groups was not significantly different (91.3% vs. 93.0%; p-value 0.622), as presented in Table 2. In other words, the failure rate were 8.8% and 7.0% in PCS group and No PCS group, respectively. Fetal delivery interval was also similar between both groups (1531 vs. 1279 min in PCS and No PCS, respectively; p-value 0.756). Kaplan-Meier curves for the rates of delivery of PCS group and No PCS group at various induction to fetal delivery time was not significantly different (Log rank test; p-value 0.664), as presented in Fig. 1. The rate of complete abortion, requiring no curettage was also not significantly different, (86.3% vs. 87.3% in PCS and No PCS, respectively; p-value 0.813), as presented in Table 2. The total misoprostol dosages were similar in both groups (1155 vs. 1164 mcg in PCS and No PCS, respectively; p-value 0.432).

**Table 2:** Termination of pregnancy outcomes

	Previous cesarean Sect. (80)	No previous cesarean Sect. (158)	P value
Fetal weight (gm)	322.44 ± 183.29	380.90 ± 220.75	0.097
Fetal delivery interval (min)	1530.72 ± 1776.63	1279.18 ± 1035.69	0.170
Placental delivery interval (min)	1486.13 ± 1601.51	1314.90 ± 1044.98	0.325
Failure to fetal delivery within 48 h .	7 (8.8%)	12 (7.6%)	0.756
Complete abortion	69 (86.3%)	138 (87.3%)	0.813
Rate of fetal delivery for all at			
12 h	27 (33.8%)	52 (32.9%)	0.897
24 h	59 (73.8%)	113 (71.5%)	0.716
36 h	67 (83.8%)	139 (88.0%)	0.367
48 h	73 (91.3%)	147 (93.0%)	0.622
> 48 h	80 (100.0%)	158 (100.0%)	-
Rate of fetal delivery for success cases (fetal delivery within 48 h) at			
12 h	27 (37.0%)	52 (35.4%)	0.814
24 h	59 (80.8%)	113 (76.9%)	0.504
36 h	67 (91.8%)	139 (94.6%)	0.427
48 h	73 (100.0%)	147 (100.0%)	-
Total misoprostol dose (mcg)	1155.0 ± 967.30	1064.56 ± 762.92	0.432

All adverse effects were comparable in both groups, except that nausea was significantly higher in No PCS group, though the incidence was very low (5.1%), as presented in Table 3. The two most common adverse effects in both groups were fever follow by chill, found in more than 40% and 30%, respectively. Uterine rupture, the most serious effect of concern, was found in 1 case, in the PCS group (1.3%), as presented in Table 3. This case was a 27-year-old woman with one previous low transverse cesarean section due to breech presentation 3 year prior to this pregnancy. TOP was performed at 20^+ 6^ weeks of gestation due to fetal holoprosencephaly. After failure of TOP within 48 h and the next day of resting period, TOP by misoprostol was restarted together with laminaria tent insertion to promote cervical ripening, followed by oxytocin infusion six hours later. However, the second course also failed to induce adequate uterine contractions. Ultrasound examination on day 6 of TOP incidentally revealed uterine rupture with fetal part out of uterine cavity. Exploratory laparotomy to remove the conceptive products and repair the cesarean rupture site was successfully performed. A total dose of misoprostol used was 2800 mcg with total time of induction for 7110 min or nearly 5 days (excluding one day of resting).

**Table 3:** Comparisons of adverse outcomes between the two groups

	Previous cesarean Sect. (80)	No previous cesarean Sect. (158)	P value
Fever	35 (43.8%)	69 (43.7%)	0.991
Chill	29 (36.3%)	54 (34.2%)	0.751
Nausea	0 (0.0%)	8 (5.1%)	0.035
Vomiting	0 (0.0%)	5 (3.2%)	0.126
Diarrhea	11 (13.8%)	26 (16.5%)	0.586
Oxytocin requirement	5 (6.3%)	21 (13.3%)	0.100
Intravenous analgesia requirement	36 (45.0%)	62 (32.9%)	0.394
Estimated blood loss (ml)			0.364
< 50 ml; n/N (%)	5/80 (6.3%)	4/158 (6.3%)	
50–499 ml; n/N (%)	74/80 (92.5%)	152/158 (96.2%)	
≥500 ml; n/N (%)	1/80 (1.3%)	2/158 (1.3%)	
Curettage required (%)	10 (12.5%)	19 (12.0%)	0.916
Uterine rupture (%)	1 (1.3%)	0 (0.0%)	0.336

**Fig. 2:**
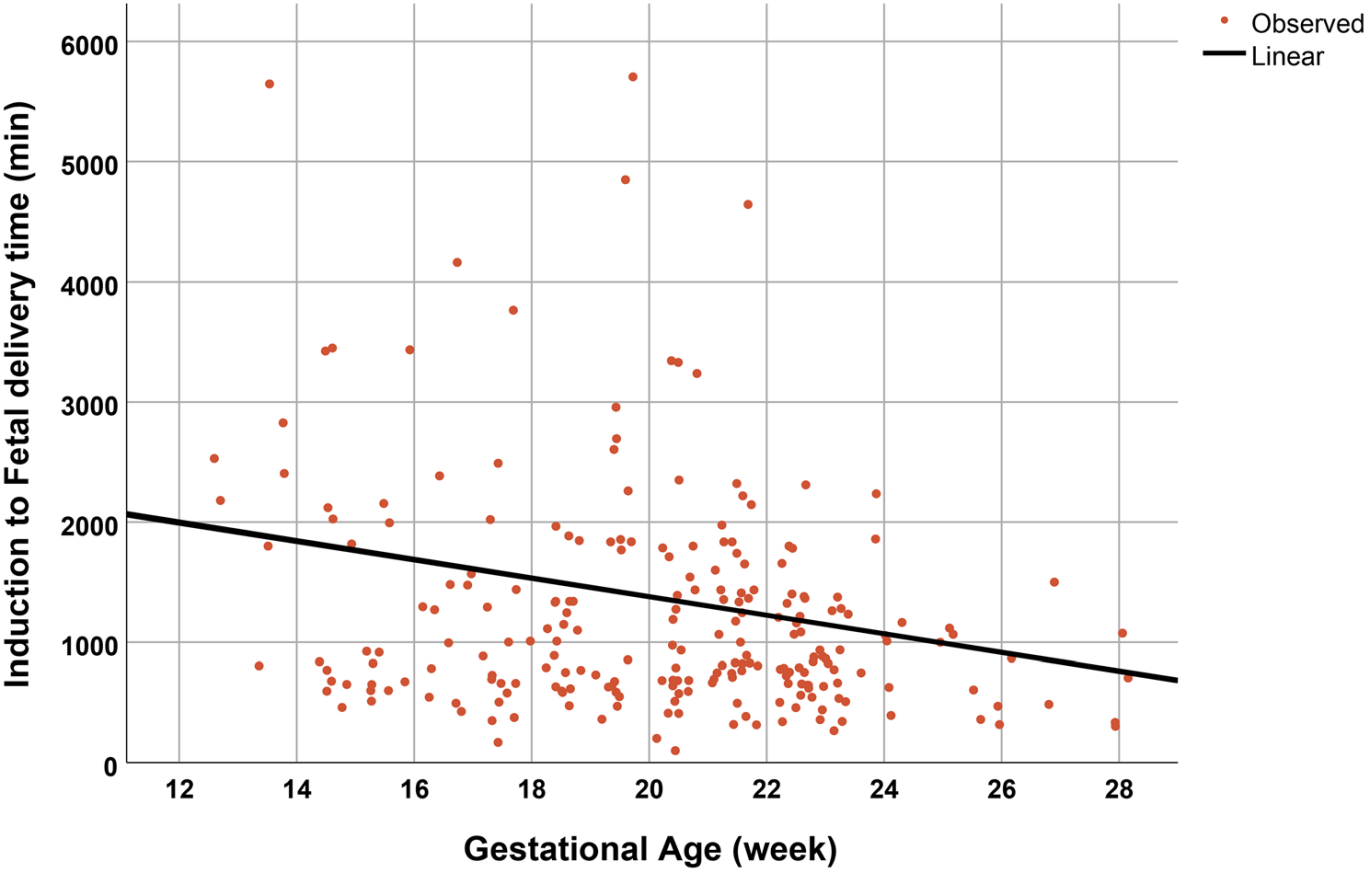
Induction to fetal delivery time as a function of gestational age (R^2^:0.043; p-value 0.005)

**Fig. 3:**
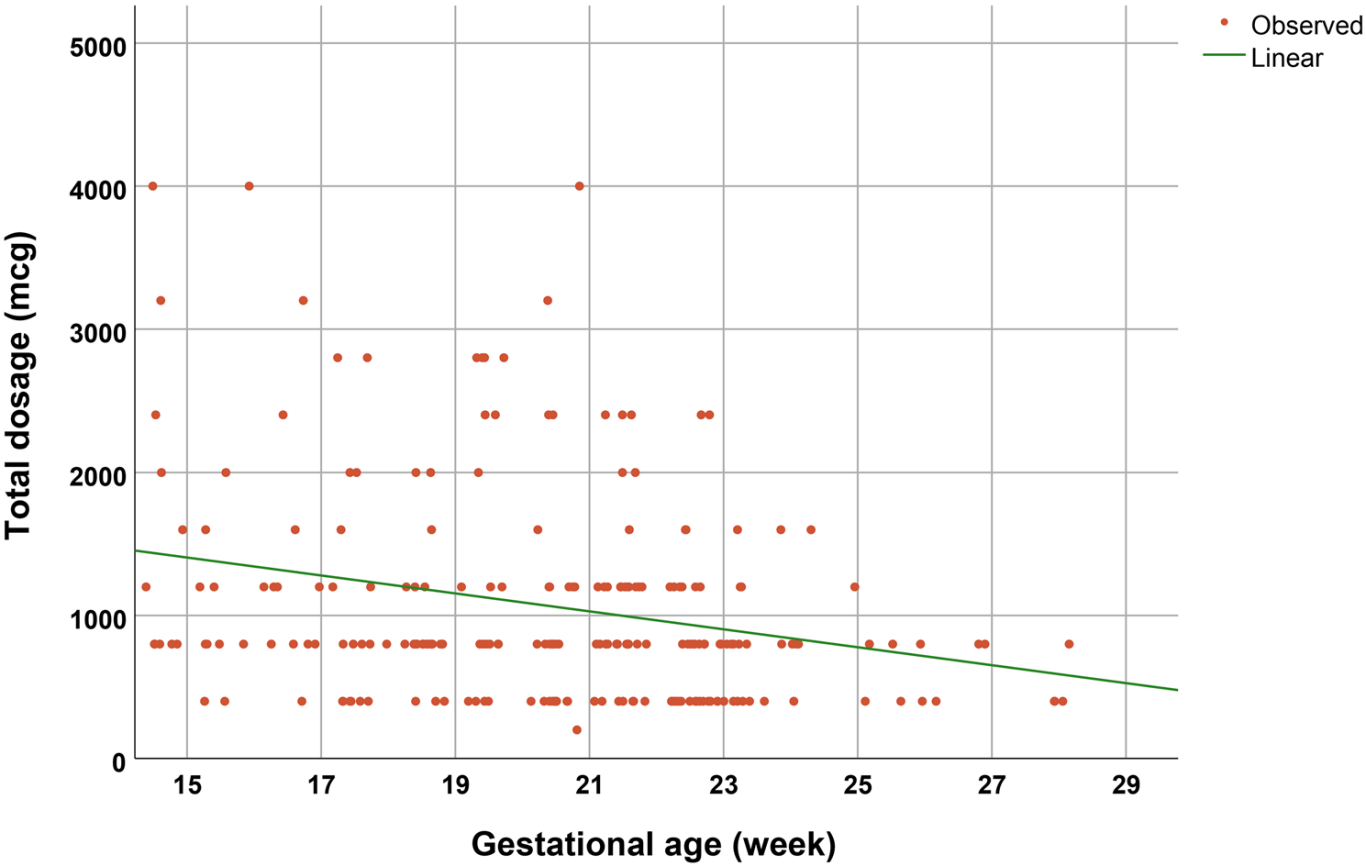
Total misoprostol dosage as a function of gestational age (R^2^: 0.060; p-value 0.001)

Univariate and multivariate analysis, performed by logistic regression analysis to evaluate the potential risk factors for successful termination, demonstrated that only gestational age was a significant independent factor for success with Odds ratio of 1.2, whereas history of PCS as well as other factors was not significantly associated with the success rate, as presented in Table 4. Note that the induction to fetal delivery time interval was significantly decreased with gestational age as a linear correlation, as presented in Fig. 2. Additionally, the total dosage of misoprostol needed for TOP was significantly decreased with gestational age, as presented in Fig. 3.

**Table 4:** Logistic regression analysis of potential risk factors for successful termination

	Univariate analysis		Multivariate analysis	
	p-value	Odds ratio (95% CI)	p-value	Odds ratio (95% CI)
Maternal Age	0.881	1.005 (0.938–1.077)	0.704	1.015 (0.939–1.098)
Pre-pregnancy BMI	0.783	1.010 (0.943–1.081)	0.901	0.996 (0.937–1.059)
Bishop score	0.383	0.808 (0.500-1.305)	0.151	0.681 (0.403–1.150)
PCS / No PCS	0.756	0.857 (0.324–2.269)	0.509	0.611 (0.142–2.630)
Nulliparous / Parous	0.623	0.790 (0.309–2.022)	0.567	0.661 (0.160–2.726)
Gestational age	0.025	1.194 (1.022–1.393)	0.016	1.219 (1.038–1.433)

## Discussion

Insights gained from this study are as follows: (1) TOP with live fetuses in the second trimester in women with PCS, using misoprostol has a highly success rate (more than 90%), comparable with that that in those with no PCS. (2) TOP with live fetuses in the second trimester in women with PCS, using misoprostol must be performed with high precaution since it carries a risk of uterine rupture in approximately 1.3%, though relatively low. (3) In TOP with live fetuses in the second trimester using misoprostol, gestational age is an independent factor for the success rate, time interval from induction to fetal delivery and total dosage of misoprostol.

According to several previous studies, TOP in the second trimester with misoprostol is safe and effective but the data on its use in women with PCS is limited and heterogeneous, in terms of fetal life status (mixed data of both live and dead fetuses [14, 15]); misoprostol-only regimens or combinations with other methods such as mifepristone [16, 17] or Foley’s catheter insertion [18, 19], and misoprostol doses (200 mcg [20, 21], 100–400 mcg [22], 200 mcg followed by 400 mcg [23], higher loading doses followed by lower doses [24–26]). Thus, to date, the consensus guidelines for TOP with PCS could not be drawn from literatures. Accordingly, WHO [13] suggests that, for those with uterine scars, the safety and efficacy of medical abortion regimens (for 12 weeks or more) is an area requiring more research, particularly the misoprostol dosage either when used in combination with mifepristone or in misoprostol-only regimens.

Based on WHO recommendations [13], misoprostol-only regimen for induced abortion at ≥ 12 weeks of gestation is 400 mcg (intravaginal, buccal or sublingual) every 3 h without loading dose. Whereas the most appropriate regimen for pregnancies with PCS is still unclear, because of safety concern, we extended the administration interval to be 6 h. Nevertheless, the success rate was still high in both groups. Additionally, the effectiveness in this study was consistent with that reported by Daskalakis et al [14]. However, the results could not be perfectly compared, since their data was relatively heterogeneous, including dead and live fetuses, cases with intact and ruptured membranes. The regimen was oral 400 mcg of misoprostol in combination with intravaginal 400 mcg misoprostol, repeated every 6 h, which was different from our study Additionally, gestational age was confined to mid-pregnancy (17 and 24 weeks), whereas our study included all gestational age of the second trimester (14 and 28 weeks).

Theoretically, misoprostol used for TOP with PCS in the second trimester might carry a higher risk for uterine rupture because of weakness at the cesarean scar. High caution must be exercised especially in cases of requirement of high doses or prolonged use, as seen in our case of uterine rupture, which needed a total dose of misoprostol 2800 mcg, with total time of induction of nearly five days. Nevertheless, the prevalence of uterine rupture in this study was not significantly different between both groups. This is likely caused by too small sample size or low power to express a significant existing difference. All cases of TOP with PCS should be considered as a very high risk for uterine rupture, as seen in 1.3% in this study, consistent with 1.3% in the study of TOP with mifepristone plus misoprostol reported by Dickinson et al. [16] and several single case reports [27–29].

We have documented important evidence that gestational age is an independent factor of success rate, induction-to-fetal delivery interval and total needed doses of misoprostol. Therefore, in considering regimens of choice for TOP in second trimester, gestational age should be taken in to account. Though this study used the same regimen for all gestational age, our results support some previous studies using higher doses in early second trimester and lower doses in late second trimester [15].

***Limitations*** of this study are as follows: (1) Though the sample size is large enough to address the primary objectives, it is relatively small to compare the rare outcomes such as uterine rupture. (2) Because pregnancy with PCS must have been pregnant before, significantly higher maternal age and higher parity among women with PCS were unavoidable. However, maternal age and parity are unlikely to affect the effectiveness of misoprostol.

***Strengths*** of this study are as follows: (1) Excluding pregnancies with a dead fetus, which could affect the effectiveness of misoprostol, and using the same regimen in both groups can enhance the reliability of comparisons. (2) The successful termination was assessed using both univariate and multivariate analysis, as well as time-event analysis (log-rank test for survival curve).

### Research implications

Our data can be served as a primary source for future studies especially meta-analysis on safety of misoprostol in terms of uterine rupture among pregnant women with previous cesarean section, requiring much more accumulated cases in literatures. Due to the findings of different response to misoprostol of the uterus in different gestational age in both TOP with and without PCS, future studies on gestational age - dependent misoprostol dosages should be conducted, for example higher dosage misoprostol regimen for pregnancy at 14–20 weeks of gestation than that for those at 21–28 weeks of gestation.

## Conclusions

While misoprostol is considered as relatively contraindicated for pregnancies with a previous uterine scar, it is much more beneficial to those women requiring TOP with a less invasive method and lower cost. We provide evidence that misoprostol is relatively safe if used with high precaution. It may be considered as one of the first line methods for TOP with previous cesarean section, especially in low-resource settings, because of its high efficacy, ease to use, low cost, and safety with precautions. However, cost-benefit or cost-effectiveness of misoprostol use in cases with previous uterine scare needs further study to compare with other available methods or other medications.

## Data Availability

The datasets analyzed during the current study are available from the corresponding author upon reasonable request.
